# Does health insurance contribute to improved utilization of health care services for the elderly in rural Tanzania? A cross-sectional study

**DOI:** 10.1080/16549716.2020.1841962

**Published:** 2020-11-25

**Authors:** Malale Tungu, Paul Joseph Amani, Anna-Karin Hurtig, Angwara Dennis Kiwara, Mughwira Mwangu, Lars Lindholm, Miguel San Sebastiån

**Affiliations:** a Department of Development Studies, School of Public Health and Social Sciences, Muhimbili University of Health and Allied Sciences, Dar es Salaam, Tanzania; b Epidemiology and Global Health, Umeå University, Umeå, Sweden; c Department of Health Systems Management, School of Public Administration and Management, Mzumbe University, Morogoro, Tanzania

**Keywords:** SAGE, access, health care use, older people, community health fund, rural, Tanzania

## Abstract

**Background**: Health care systems in developing countries such as Tanzania depend heavily on out-of-pocket payments. This mechanism contributes to inefficiency, inequity and cost, and is a barrier to patients seeking access to care. There are efforts to expand health insurance coverage to vulnerable groups, including older adults, in Sub-Saharan African countries.

**Objective**: To analyse the association between health insurance and health service use in rural residents aged 60 and above in Tanzania.

**Methods**: Data were obtained from a household survey conducted in the Nzega and Igunga districts. A standardised survey instrument from the World Health Organization Study on global AGEing and adult health was used. This comprised of questions regarding demographic and socio-economic characteristics, health and insurance status, health seeking behaviours, sickness history (three months and one year prior to the survey), and the receipt of health care. A multistage sampling method was used to select wards, villages and respondents in each district. Local ward and hamlet officers guided the researchers in identifying households with older people. Crude and adjusted logistic regression methods were used to explore associations between health insurance and outpatient and inpatient health care use.

**Results**: The study sample comprised 1,899 people aged 60 and above of whom 44% reported having health insurance. A positive statistically significant association between health insurance and the utilisation of outpatient and inpatient care was observed in all models. The odds of using outpatient (adjusted OR = 2.20; 95% CI: 1.54, 3.14) and inpatient services (adjusted OR = 3.20; 95% CI: 2.46, 4.15) were higher among the insured.

**Conclusion**: Health insurance is a predictor of outpatient and inpatient health services in people aged 60 and above in rural Tanzania. Further research is needed to understand the perceptions of both the insured and uninsured regarding the quality of care received.

## Background

For many years, health care systems in low- and middle-income countries (LMICs), including Tanzania, have depended heavily on out-of-pocket payments (OOP) for financing health care services [1,2]. There is an extensive body of evidence describing how OOP mechanisms have contributed to inefficiencies, inequities and higher costs, and have become a barrier for patients and families seeking to access affordable health care [3,4]. This has also led to a failure of countries to sustain sufficient funding for health care. Attempts to change health care financing systems are ongoing in many sub-Saharan African countries [5,6]. These efforts aim to improve the protection and utilisation of health care, particularly for vulnerable sub-groups [7–9]. Over the last two decades, many African countries have been looking at the possibilities of introducing and expanding health insurance (HI) coverage, installing effective exemption mechanisms for those unable to pay, and improving tax collection and allocation to health care [10–12].

This pattern is in line with the World Health Organisation (WHO) policy to include HI as a tool for financing health care in all countries [3,6,13]. The main reason is that pre-payments and financial risk pooling within HI can guarantee the utilisation of equitable and quality health care services to the insured at affordable rates depending on their willingness, ability to pay and the expected returns [11]. Globally, HI takes two main forms – either mandatory or voluntary. For instance, in some countries such as Vietnam and the Philippines, HI has taken the mandatory enrolment path, whereas voluntary insurance mechanisms that include a mix of private and community-based health care financing, are operating in Ghana, Senegal and Rwanda [14,15]. The design and implementation of HI and the utilisation of health services, varies across countries. Evidence from studies conducted to investigate whether public HI increases access to health care services in LMICs shows that HI not only increases access and utilisation but also provides financial protection and improves the population’s health status [16,17]. In order to achieve universal health coverage (UHC) in many poor countries, introducing and expanding the coverage of state owned and financed insurance schemes is necessary in order to reach vulnerable populations [18,19].

Social health insurance (SHI), tax-based insurance or a mix of the two, have historically been common insurance approaches in some countries including South Africa, Benin, Brazil, Mexico and Thailand [20]. Whereas SHI can be voluntary or mandatory and focuses on providing coverage to rurally based populations and the informal sector, tax-based systems are mainly financed through tax revenues and are accessible to all citizens. Many LMICs have implemented both forms in attempting to accelerate the achievement of universal health coverage (UHC). Nevertheless, public funded HI has also been criticised for not improving the quality of services or the availability of medicines and for increasing waiting times [21]. A study from Ghana showed that HI did not protect vulnerable groups equally from financial risk, with the uneducated, poor and self-employed living far from hospitals and experiencing relatively lower reductions in catastrophic OOP medical spending compared with their counterparts [22]. Similarly, other studies from India and Tanzania have reported how public-owned HI failed to achieve full financial protection and decrease OOP among enrolled household members [23,24]. In addition, most African countries have implemented community-based HI, which only covers a specific city or region, making sustainability a challenge due to limited numbers of enrolees [25].

Health insurance was introduced in Tanzania with the long-term goal of achieving UHC [26]. Two prepaid governmental systems, the Community Health Fund (CHF) and the National Health Insurance Fund (NHIF), were implemented in 1996 and 2001 respectively. Around 24% of the Tanzanian population is covered under the CHF and 9% under the NHIF. The CHF started as a pilot scheme in the Igunga district, and was rolled out later to other parts of the country. The scheme was seen as a possible mechanism for granting access to basic, affordable, quality health care services to the rural population [11]. Membership in the CHF remains voluntary, and households contribute the required amount set by the district/municipal CHF board [27]. Each member is given a renewable health access card that entitles the household to a basic package of health care services within the district’s health facilities throughout the year [11]. Initially, the NHIF was introduced to cover health care services for central government employees, but in 2013, it was expanded to the informal sector. However, although the NHIF remains compulsory for government employees, it has remained voluntary to members of the informal sector. These two schemes operate in parallel within each district. There is no risk pooling across the schemes. People who have retired from the public sector who had already joined the NHIF continue to be covered until their death, whereas those who were not members can voluntarily join and pay the required premiums, which are between USD 23.79–36.23 per year for the NHIF and between USD 2.18–6.55 per year for the CHF. These amounts are set by the districts as the levels required for complete coverage [28]. This insurance guarantees older people access to health care services including routine examinations and treatments for both outpatient and inpatient care.

Currently, all public and some accredited charity and private facilities provide health care services (primary, secondary and tertiary health care) that are available to all insured members, regardless of age and locality [8,11]. These services include outpatient consultations, medicines, surgical services, inpatient services, physiotherapy and rehabilitation services, eye and optical services, oral health services, retirees’ health benefits and medical/orthopaedic appliances [10,29,30]. The CHF members can only access these services at public or at accredited private facilities within their respective district. Those covered by the NHIF have wider access that extends beyond their area of residence. There are both public and private facilities accredited by the scheme at all levels of service within the country.

There is evidence that the CHF reaches a large number of low-income households who would otherwise have no financial protection against the costs of illness [11,31,32]. However, research has also shown that where the CHF is practised, health benefits are not evenly distributed. The poorest segment of the population receives a lower share of health care benefits relative to their needs [10].

Since the introduction of HI in Tanzania, a number of studies have been conducted to investigate the extent to which HI has benefited the population. Some of these studies have shown that HI increased the level of outpatient care and influenced the places where the insured went for care [10,26]. A study that focused on assessing the determinants of HI participation among informal sector workers in rural Tanzania, showed that HI is widely accepted as it enhances the affordability, accessibility and quality of care. However, the results showed that HI was challenged by mistrust and a lack of knowledge about the schemes [11]. A subsequent study in Tanzania explored the factors and challenges that influence the accessibility of health care delivery to HI members [33,34]. It revealed that poor quality of services, prolonged registration processes and health care units’ preference for patients who paid cash were among the challenges facing HI fund members. Many other studies have focused on evaluating the performance of HI in terms of enrolment, financial management and sustainability, as well as by patients’ perceptions of services offered under HI [35,36]. However, to our knowledge, no studies in Tanzania have specifically dealt with older adults as a population sub-group. Given the ageing of the rural population and associated health system challenges, it is important to explore the role of HI in this population. In order to address this evidence gap, this study analyses the association between HI and health service use in rural residents aged 60 and above in Tanzania. This work is of relevance to government efforts to establish UHC in Tanzania in the near future. The findings will also support service users and providers as well as inform policy and decision-making at the district and national levels to improve and promote health care access, utilisation and coverage for older rural residents of Tanzania.

## Methods

### Study design

A population-based cross-sectional designed study was conducted to collect data from the households of insured and uninsured people, aged 60 and above, between July and September 2017.

### Study setting

The study was conducted in the Nzega and Igunga Districts in in the Tabora Region. Both are rural districts in the central part of Tanzania. Tabora is one of 31 administrative regions. According to the 2012 population census, there are 2.3 million inhabitants of whom 901,979 live in the Igunga and Nzega Districts [37]. In the two districts, there are approximately 50,547 people aged 60 years and older. The Nzega and Igunga Districts were appropriate places for this study because around 92% of the people aged 60 and above live in these districts. With regard to HI, the Tabora region has 20,326 insured persons. The two districts have a total of 5,571 people aged 60 and above with mainly NHIF and CHF. This corresponds to 13% in Igunga and 20% in Nzega of the total insured population. The Igunga district was the first district in Tanzania to implement CHFs in 1996. The two districts were chosen for logistical reasons, as they are neighbouring rural districts with a high proportion of elderly people with HI. Both primary and secondary health care facilities are available for residents of all ages, regardless of HI. An insured older person can access a range of free outpatient and inpatient services and medications obtained at public and private accredited facilities. These services also include physiotherapy rehabilitation, surgery, optical and dental services [10,11].

### Data collection

The target group was people aged 60 years and older. A pre-tested household questionnaire based on the instrument used in World Health Organization Study on global AGEing and adult health (WHO-SAGE) was used. The questions captured information on demographic and socio-economic characteristics, health and insurance status, health seeking behaviours, sickness history (previous three or twelve months prior to the survey) and whether health care was sought and received from accredited health facilities [38].

### Sample size and sampling techniques

We used a multistage sampling method to select wards and villages from each district. In the first stage, a convenience sampling technique was used to randomly select seven wards (about one fifth of the total number) from each district. In the second stage, 58 villages were randomly selected using a lottery method. In the third stage, hamlet officers helped in the selection of 25 to 44 households (with occupants aged 60 and above) from each village. The number of households was dependent on village size. In this study, both males and females had an equal chance of participation. In cases where one household had two possible respondents, a lottery method was used to select one survey participant. All those who were invited agreed to participate in the survey.

The required sample size was estimated at 733. This was based on an estimated prevalence of health care utilisation (the outcome) of 40%, a design effect of 2, a 95% confidence interval and 80% power. We doubled the sample size in order to improve representativeness.

### Variables

Health care utilisation is associated with HI, health needs, and socioeconomic and demographic factors. There were two outcome variables – outpatient health care utilisation and inpatient health care utilisation. They were derived from answers to the following questions: (i) ‘Over the last three months, did you receive any health care, not including an overnight stay in hospital or long-term care facility?’ (Yes or No) and (ii) ‘In the last 12 months, have you ever stayed overnight in hospital?’ (Yes or No).

The possession of HI was determined by asking if respondents currently held HI (Yes or No). This reference to HI included public HI (i.e. NHIF or CHF) and other private HI. Respondents were also asked to provide the length of time they have had HI (less than or equal to one year, more than one year).

The need for health care was captured in the following variables: (i) sex (female/male), (ii) age (categorised into 60–69, 70–79, >79), (iii) self-rated health, that is, *‘How would you assess your general state of health today?’* (very good, good, moderate, bad, very bad), (iv) reasons for outpatient care: *‘Did you experience any disease(s) in the last three months?’* (no diseases, one disease, more than one disease), and (v) reasons for inpatient care: *‘Did you experience any disease(s) in the last 12 months?’* (no disease, one disease, more than one disease).

Socioeconomic and demographic variables were considered as enablers of health care access: (i) marital status, which included those who were married (i.e. currently married and cohabiting) and currently non-married (i.e. widows, separated, never married and divorced); (ii) education: *‘What is the highest level of education that you have completed?’* (low = less than or equal to primary education; medium = secondary education; and high = above secondary education); and (iii) income: *‘What is the monthly total income of your household?*’ (below = those with less than or equal to USD 22.50; above = more than USD 22.50). These income levels were defined based on the Tanzanian basic need poverty line (2011/2012) estimation of 36,482Tsh per adult per month which is roughly equivalent to USD 22.50 [39].

### Data analysis

A descriptive analysis of the study sample was conducted. No missing values were found in the data. Logistic regression was used to assess association between HI and health care utilisation. Four models were constructed for each of the two outcome variables – i.e. outpatient or inpatient health care use in the previous three or twelve months. Model 0, the crude model, includes only the independent variable, HI. Model 1 also includes demographic (sex, age) and socioeconomic variables (marital status, education and income). Model 2 includes HI and the self-reported health variables. Model 3 includes HI and all other covariates. All regression models were adjusted for the clustered nature of the observations in the villages and potential correlation within villages using the Stata software version 15.

### Ethics

Ethical clearance was obtained from the Muhimbili University of Health and Allied Sciences (MUHAS-Research Review Board) in May 2017 (reference number 2017–05-24/AEC/Vol.XII/70). Permission for field data collection was granted by the District Executive Directors of the Igunga and Nzega districts. Informed written consent was obtained from participants and verbal consent was obtained from those who could not read or write. Before seeking consent, participants were fully informed about the research objective and their rights to participate or withdraw from the study.

## Results

### Characteristics of the respondents

As shown in Table 1, the final study sample comprised 1,899 people aged 60 years and older. Out of these respondents, 51.0% were women and 48.7% were men. Regarding HI, only 44.3% of respondents reported having HI and of these, 94% belonged to either CHF or NHIF and 6% had private HI. Over half of the respondents (58.7%) were between 60 and 69 years old, 26.7% were between 70 to 79 years old, and the remaining 14.9% were above 80 years old. About 71% of the women were currently not married (i.e. widows, separated, never married or divorced), whereas about 61% of the men were married. With regard to health status, 28% reported their self-rated health as being ‘good’, 48% as ‘moderate’ and 24% as ‘bad’. During the prior three months, 28% of the respondents reported no disease, whereas 52% and 20% reported to have one disease and more than one disease, respectively. In comparison, during the past 12 months, 67% reported no disease, 25% reported to have one disease and 8% reported more than one disease. Regarding education, 59% had no education, whereas 41% of the respondents had a formal education in the following categories: 47% low-level, 46% medium-level and only 7% high-level. About 69% of participants reported a household income of less or equal to USD 22.50 per month. Among the respondents, 76.8% had used outpatient health care services in the three months prior to the survey, and 39.2% had attended inpatient care in the past 12 months.

**Table 1.** Health insurance ownership, health need and demographic and socioeconomic characteristics of participants, stratified by sex (N = 1899)

	Men	Women	Total (%)
*Health Insurance*			
No	529 (56.88%)	529 (54.59%)	1,058 (55.71)
Yes	401 (43.12%)	440 (45.41%)	841 (44.29)
Need variables			
*Age*			
60–69	517 (55.59%)	594 (61.3%)	1,111 (58.5)
70–79	264 (28.39%)	242 (24.97%)	506 (26.65)
>79	149 (16.02%)	133 (13.73%)	282 (14.85)
*Self-rated health*			
Good	283 (30.43%)	244 (25.18%)	527 (27.75)
Moderate	436 (46.88%)	484 (49.95%)	920 (48.45)
Bad	211 (22.69%)	241 (24.87%)	452 (23.8)
*Diseases in the last three months*			
No disease	265 (28.49%)	268 (27.66%)	441 (28.07%)
Yes, 1 disease	471 (50.65%)	511 (52.73%)	982 (51.71%)
Yes, >1 disease	194 (20.86%)	190 (19.61%)	384 (20.22%)
*Diseases in the last 12 months*			
No disease	627 (67.42%)	642 (66.25%)	1,269 (66.82%)
1 disease	228 (24.52%)	251 (25.9%)	479 (25.22%)
>1 disease	75 (8.06%)	76 (7.84%)	151 (7.95%)
Demographic and socioeconomic variables			
*Marital status*			
Married	569 (61.18%)	279 (28.79%)	848 (44.66)
Currently not married	361 (38.82%)	690 (71.21%)	1,051 (55.34)
*Education*			
None	473 (50.81%)	654 (67.49%)	1,127 (59.35)
Low	204 (21.91%)	158 (16.32%)	362 (19.06)
Middle	219 (23.52%)	138 (14.26%)	357 (18.80)
High	35 (3.76%)	18 (1.86%)	53 (2.79)
*Income*			
≤ 22.5 USD	629 (67.63%)	673 (69.45%)	1,302 (68.56)
> 22.5 USD	301 (32.37%)	296 (30.55%)	597 (31.44)
Outcome variables			
*Outpatient care used last 3 months*			
No	223 (23.98%)	218 (22.50%)	441 (23.22)
Yes	707 (76.02%)	751 (77.50%)	1,458 (76.78)
*Inpatient care used last12 months*			
No	581 (62.47%)	574 (59.24%)	1,155 (60.82)
Yes	349 (37.53%)	395 (40.76%)	744 (39.18)

### Regression analysis

Tables 2 and 3 present the results of the logistic regression analyses indicating the crude and multivariable estimates of association between HI and both outpatient and inpatient health care access, in the presence of socioeconomic, demographic and need variables.

**Table 2.** Bivariate and multivariable logistic regressions of the association between HI and outpatient services (n = 1899)

	Model 0	Model 1	Model 2	Model 3
	OR (95% CI)	OR (95% CI)	OR (95% CI)	OR (95% CI)
*Health Insurance*	3.03 (2.38, 3.84)*	3.02 (2.06, 4.43)*	2.18 (1.53, 3.11)*	2.20 (1.54, 3.154)*
*Sex*				
Men		1		1
Women		0.94 (0.69, 1.29)		0.97 (0.71, 1.34)
*Age*				
60–69		1		1
70–79		0.88 (0.67, 1.15)		0.87 (0.66, 1.13)
>79		1.25 (0.90, 1.72)		1.10 (0.77, 1.57)
*Marital status*				
Married		1		1
Currently not married		1.27 (0.91, 1.77)		1.34 (0.95, 1.90)
*Education*				
None		1		1
Low		1.38 (0.94, 2.03)		1.31 (0.88, 1.96)
Middle		1.74 (1.15, 2.65)*		1.73 (1.12, 2.67)*
High		0.78 (0.49, 1.77)		0.85 (0.55, 1.31)
*Income*				
≤ 22.50 USD		1		1
> 22.50 USD		1.40 (0.91, 2.17)		1.40 (0.89, 2.19)
*Diseases in the last three months*				
No disease			1	1
1 disease			4.91 (3.16, 7.63)*	4.89 (3.112, 7.66)*
>1 disease			6.40 (2.35,17.45)*	6.27 (2.30, 17.11)*
*Self-rated health*				
Good			1	1
Moderate			0.93 (0.65, 1.33)	0.94 (0.65, 1.35)
Bad			1.52 (0.86, 2.69)	1.51 (0.85, 2.70)

* p-value <0.05; OR: Odds Ratio; CI:-Confidence Interval

**Table 3.** Bivariate and multivariable logistic regressions of the association between HI and inpatient services (n = 1899)

	Model 0	Model 1	Model 2	Model 3
	OR (95% CI)	OR (95% CI)	OR (95% CI)	OR (95% CI)
*Health Insurance*	3.59 (2.76, 4.67)*	3.60 (2.74, 4.73)*	3.18 (2.46, 4.11)*	3.20 (2.46, 4.15)*
*Sex*				
Men		1		1
Women		0.84 (0.67, 1.04)		0.83 (0.66, 1.04)
*Age*				
60–69		1		1
70–79		0.96 (0.78, 1.18)		0.99 (0.80, 1.22)
>79		1.50 (1.17,1.92)*		1.47 (1.14, 1.90)*
*Education*				
None		1		1
Low		1.17 (0.82, 1.67)		1.09 (0.76, 1.55)
Middle		1.04 (0.71, 1.53)		0.98 (0.67, 1.44)
High		0.76 (0.45, 1.29)		0.77 (0.45, 1.31)
*Marital status*				
Married		1		1
Currently not married		0.87 (0.70, 1.09)		0.84 (0.67, 1.05)
*Income*				
≤ 22.50 USD		1		1
> 22.50 USD		1.02 (0.70, 1.09)		0.98 (0.73, 1.32)
*Diseases in the last 12 months*				
No disease			1	1
1 disease			2.66 (1.89, 3.75)*	2.66 (1.87, 3.77)*
>1 disease			3.02 (1.90, 4.81)*	2.97 (1.86, 4.73)*
*Self-rated health*				
Good			1	1
Moderate			0.94 (0.72, 1.24)	0.92 (0.70, 1.21)
Bad			1.13 (0.80, 1.60)	1.10 (0.77, 1.56)

* p-value <0.05; OR: Odds Ratio; CI: Confidence Interval

### Outpatient services

A positive statistically significant association between HI and the utilisation of outpatient care was observed in all regression models. See Table 2. In the crude model the odds of visiting health care were three times higher for those with HI compared to those without HI (OR = 3.03; 95% CI: 2.38, 3.84). The effect attenuated after successively adjusting for demographic and socioeconomic variables (Model 1) and health need variables (Model 2) with Model 3 showing that people with HI were over twice as likely to use outpatient services (OR = 2.20; 95% CI: 1.54, 3.14). Respondents who reported having had a disease in the three months prior to the survey were significantly more likely to use outpatient services. See Models 2 and 3.

### Inpatient services

As for outpatient health care, there was a positive statistically significant association between HI and the utilisation of inpatient care in all regression models (Table 3). In the crude model, the odds of visiting health care were more than three times higher (OR = 3.59; 95% CI: 2.76, 4.67) in those with HI compared to those without HI. This association remained after successively adjusting for demographic and socioeconomic variables (Model 1) and health need variables (Model 2) (OR = 3.20; 95% CI: 2.46, 4.15). As was the case for outpatient services, respondents who reported having had a disease in the three months prior to the survey were significantly more likely to use inpatient services.

## Discussion

This study analysed association between HI and the use of outpatient and inpatient health care services by people aged 60 and above in rural areas of Tanzania. Over 40% of study respondents reported HI. Health insurance was significantly associated both outpatient and inpatient service utilisation after adjusting for socioeconomic and demographic factors and health care need. This suggests that HI is an important enabler of access to health care.

The results are consistent with other studies conducted in Tanzania and elsewhere that have investigated the effect of HI on access to care [26,40–43]. For example, the study by Chomi et al. [26] which examined health seeking behaviour and utilisation by members of different insurance schemes in Tanzania, showed that, after adjusting for health needs, insured individuals were more likely to seek care and less likely to experience delays. Another study that examined the effects of fragmented risk pooling and health-care-seeking behaviour among members of CHFs and NHIFs in Tanzania suggested that the insured stand a better chance of accessing care and experience less delays compared to the uninsured [44]. A study by Msuya et al. [45] found that people with CHF membership in Tanzania had a greater chance of being financially protected against health shocks than non-members. In addition to these studies in Tanzania, similar findings have been reported in studies in Ghana and Senegal [46–48] where HI was associated with greater access to health care services among the older compared to the younger population group and also non-health insurance members. In our study, factors such as sex, education and marital status showed no statistically significant association with access to both outpatient and inpatient health services before and after adjusting for need variables. Our results differed from the findings of some earlier studies that document patient characteristics, such as age and sex, influencing the demand and utilisation of health care services among older adults. For example, in both Ghana and Brazil, insured older female patients used more outpatient and inpatient services than their male counterparts [49,50].

Results from this study also seem to suggest a horizontal and vertical equity in access both for outpatient and inpatient services. The principle of horizontal equity emphasises the importance of equal access to health care for equal need, independent of socioeconomic factors [51]. This can be seen in model 3 (Tables 2 and 3) where the association between socioeconomic factors and health care use remained after adjusting for disease and self-rated health. Although little is known about health care equity in Tanzania, our findings are somewhat similar to those of Mtei et al. [10], which indicated a fairly even equal distribution of health care benefits among the members of the community health fund in the country. Our findings differ from those in many African countries, and other countries like China [52] and India [53]. For example, studies conducted in Ghana in the population aged 50 and above show a pro-rich inequity whereby those with high incomes accessed more outpatient care than those with low incomes for the same health needs [54,55,56].

Vertical equity refers to receiving different health care when needs are unequal [51]. This is observed in model 3 in Tables 2 and 3 whereby having more diseases was associated with greater health care use even after adjusting for socioeconomic variables. Although no comparable studies exist in Tanzania, this finding is similar to some other studies from LMICs. For example, Sandhi et al. [50] reported that poor health conditions increased the usage of both outpatient and inpatient health care services in patients older than 60 in Brazil. This situation has also been reported in India, where people aged 60 and above with poorer health accessed more outpatient care than their counterparts with less serious health problems [57].

The equity of access observed here may reflect the Tanzanian government’s policies that have focused on improving access to health care across all segments of the population. Some examples include the introduction of the waivers and exemptions policy in 1994, the centralisation of the control and distribution of medicines through the Medical Stores Department, and the implementation of an -specific window for drug deliveries for older persons in each health facility [12,58,59].

## Methodological considerations

To ensure consistency of the collected information and to allow comparison with other studies, the standardized SAGE questionnaire was used [60–63]. Pilot testing and training of the research assistants on data collection minimised the risk of misinterpretation by the interviewers and which may have led to respondent bias. The recruitment of local research assistants conversant with the language and culture of the study participants may have contributed to the high response rate (95%). In order to minimise bias, the questionnaire was pilot tested in a randomly selected group of people aged 60 and above. We also asked to see the insurance cards and hospital identification numbers from a randomly selected number of respondents. It is important to note that we could not distinguish the type of HI held.

However the use of a self-reported survey created the possibility for recall bias, for instance in regard to the socioeconomic, health care use and health-related variables. It is also possible that regarding inpatient care, some respondents did not report a disease experienced in the last 12 months if they had already reported it in relation to the previous three months. However, when additional analyses were restricted to the three-month period variable in inpatient care, the results pointed out in the same direction of the main findings. It is also possible that some participants with HI opted to pay for their health care services instead of using their HI benefits. However it was not possible to assess the extent of this potential misclassification. Further recall bias may have influenced validity and reliability of the study findings.

We acknowledge that selection bias may have been an issue as participants were selected with the help of hamlet officers. Three further issues need mention in regard to HI. First, adverse selection is the effect that occurs when the people most attracted to purchasing HI are those who are the most costly to insure. Although this is not relevant in the Tanzanian context, it may still be relevant if, for instance, a greater proportion of sicker persons are enrolled or those insured had better availability for health facilities in their district. The possibility of moral hazard, the overuse of free health care once people have obtained HI, is also a consideration. This was addressed in part by adjusting for health need in the analysis. We also acknowledge that the variation of CHF enrolment across different districts [30] may limit the generalisability of these findings.

## Conclusion

Almost half (44%) of the participants in this study were insured, and a positive statistical association between health insurance and use of out- and inpatient services was found. These findings support the national goal of introducing HI to protect individuals and households from catastrophic health expenditures. Both horizontal and vertical equity in the utilisation of outpatient and inpatient care services was also observed. These results suggest the importance of health insurance in providing financial protection to older adults, particularly those residing in rural areas. Extending insurance coverage to vulnerable sub-populations can improve health and equity and reduce OOP. Further research is needed to understand the perceptions of insured and uninsured elderly people regarding the quality of the care they receive.

## Paper context

We analysed association between health insurance and the use of outpatient and inpatient health care by adults aged 60 and above living in rural areas of Tanzania. The insured were more likely to use health care services even after adjusting for socioeconomic, demographic and need factors. The findings support the national goal of extending health insurance to protect individuals and households from catastrophic health expenditures. Increasing insurance coverage to vulnerable groups, such as older residents and those in rural areas, can improve health and equity of access and possibly reduce out- of -pocket payments.
